# Woodward procedure with intraoperative neuromonitoring for Sprengel deformity: a retrospective study with a mean 5-year follow-up

**DOI:** 10.3389/fped.2025.1541132

**Published:** 2025-06-27

**Authors:** Li Zhang, Haoqi Cai, Yangbo Huang, Mengting Yang, Shi Huang, Mingyuan Miao, Haiqing Cai, Zhigang Wang

**Affiliations:** ^1^ Department of Pediatric Orthopedics, Shanghai Children’s Medical Center, Shanghai Jiao Tong University School of Medicine, Shanghai, China; ^2^ Department of Neurosurgery, Shanghai Children’s Medical Center, Shanghai Jiao Tong University School of Medicine, Shanghai, China; ^3^ Department of Pediatric Orthopedics, Shenyang Children’s Hospital, Shanghai, China

**Keywords:** sprengel deformity, intraoperative neuromonitoring, clavicle osteotomy, brachial plexus injury, somatosensory evoked potentials, motor evoked potentials

## Abstract

**Background:**

The Woodward procedure was designed to correct Sprengel deformity (SD) while brachial plexus injury remains a critical complication. This study aimed to assess the effectiveness of the Woodward procedure with clavicle osteotomy and intraoperative neuromonitoring (IONM) in enhancing postoperative functional outcomes, cosmetic appearance, and nerve injury prevention.

**Methods:**

We retrospectively reviewed the records of patients who underwent the Woodward procedure with clavicle osteotomy and IONM for Sprengel deformity at our institution between January 2013 and May 2023. Patient demographics, clinical and cosmetic outcomes before and after surgery, and complications were analyzed.

**Results:**

A total of 36 patients (female: male = 16:20) with a mean age of 4.1 ± 1.5 years were included, with a mean follow-up of 5.6 years. Intraoperatively, no IONM alerts occurred. At the latest follow-up, shoulder abduction improved by an average of 74°, and Cavendish grade improved from grade 3 or 4 preoperatively to grade 1 or 2. Radiographic analysis showed Rigault grade improvement from grade 3 to grade 1 or 2. Aside from a single instance of delayed wound healing and one case of hypertrophic scarring, no brachial plexus injuries or long-term complications were observed.

**Conclusions:**

Woodward procedure combined with clavicle osteotomy and IONM for SD patients is associated with significant improvement in postoperative functional outcome, cosmetic appearance, low risk of complications and high levels of satisfaction.

## 1 Introduction

Sprengel deformity (SD) is a rare congenital abnormality characterized by abnormal elevation and inward-rotated of the scapula, accompanied by rigid periscapular tissues (1). This results in impaired shoulder abduction and a notable cosmetic deformity. The severity of Sprengel deformity is graded using the Cavendish classification (2), which ranges from very mild to severe, with surgical intervention typically indicated only for severe cases. The Woodward procedure remains a cornerstone for the surgical correction of Sprengel deformity, particularly in cases of severe deformity (Cavendish grade III and IV) (3). It involves the release and repositioning of the muscles attached to the medial border of the scapula, along with the resection of any omovertebral bone. Previous studies have reported satisfactory functional and cosmetic outcomes following the Woodward procedure (4). However, risks of brachial plexus injury (BPI) remain a significant concern during Sprengel deformity correction.

BPI can occur when the brachial plexus and subclavian artery become compressed between the clavicle and the first rib during the inferior repositioning of the scapula (5). This injury poses a risk of transient palsy or permanent neurological impairment, such as motor weakness and sensory deficits in the affected arm. This risk is particularly heightened in older children, whose reduced soft tissue flexibility and compliance exacerbate the likelihood of nerve injury (6, 7). Reported BPI rates range from 0 to 12.5%, with transient brachial plexus palsy predominantly occurring in cases without clavicle osteotomy (8, 9). Although spontaneous nerve regeneration generally resolves transient palsy within 1–7 months postoperatively, BPI remains a critical concern due to the potentially devastating consequences if nerve regeneration fails (10). Therefore, beyond standard measures such as thorough preoperative planning, meticulous intraoperative dissection, minimizing nerve traction, and preserving vascular supply, further precautions-such as concomitant clavicle osteotomy and intraoperative neuromonitoring-have been recommended to prevent BPI (11).

Clavicle osteotomy, when used as an adjunct to the Woodward procedure, plays a pivotal role in optimizing both cosmetic and functional outcomes, especially in cases of severe shoulder girdle deformity (8). By adjusting the length or position of the clavicle, it helps restore proper anatomical alignment of the shoulder girdle. It creates more space for the scapula's repositioning, facilitating its intraoperative downward movement and rotation, thereby enhancing both postoperative appearance and shoulder function. Importantly, previous studies reported clavicle osteotomy can also help reduce the risk of BPI by relieving compression of the brachial plexus during the inferior displacement of the scapula (12).

Intraoperative neuromonitoring (IONM) has been widely utilized in surgeries involving critical neural structures, such as neurosurgery (13), spinal surgeries (14), thyroidectomy (15), where it has been shown to reduce the risk of nerve injury and improve patient outcomes. Given its proven effectiveness in these fields, IONM has potential to enhance the safety of the Woodward procedure by providing real-time feedback on nerve function, thereby minimizing the risk of BPI and thereby contributing to improved functional and cosmetic outcomes. However, limited data are available on IONM's application specifically in Sprengel deformity correction (11). In the present study, we employed both clavicle osteotomy and intraoperative neuromonitoring with somatosensory evoked potentials (SEP) and motor evoked potentials (MEP) during the Woodward procedure for SD correction, aiming to evaluate their feasibility and efficacy in preventing brachial plexus injury while enhancing functional and cosmetic outcomes.

## 2 Material and methods

### 2.1 Patients demography

This retrospective study was approved by the medical ethics committee of our institution. Written informed consent was obtained from the legal guardians of all participants. A total of 36 patients (16 females and 20 males) with Sprengel deformity who underwent the Woodward procedure between January 2013 and May 2023 were retrospectively analyzed. Preoperative data, including the Cavendish classification (based on the cosmetic appearance of the scapula), the Rigault scale (based on radiologic evaluation), and the active shoulder abduction function, were prospectively recorded and compared with postoperative results. Additional details such as the presence of an omovertebral bone, parents' satisfaction, and postoperative complications were also documented. All patients underwent surgery involving clavicular osteotomy followed by the Woodward procedure. Intraoperative neuromonitoring (IONM) reports were reviewed for significant changes during the procedure. Postoperatively, all patients fully recovered from anesthesia, and an immediate neurological examination was performed to evaluate motor strength and sensory function for any deficits. To assess vascular integrity, the radial pulse was palpated intraoperatively and postoperatively, while finger oxygen saturation, skin color, and temperature were closely monitored after the procedure. Intraoperative and postoperative complications were noted. The minimum follow-up period was 1 year.

### 2.2 Surgical steps

All surgeries were performed by two surgeons, Dr. Zhigang Wang and Dr. Haiqing Cai. After administering general anesthesia, we initially performed clavicle osteotomy with the patient in the supine position, followed by the Woodward procedure in the prone position. For the clavicle osteotomy, the patient was first positioned supine. A skin-crease incision was made along the middle third of the ipsilateral clavicle region. Careful dissection down to the clavicle was performed, preserving the periosteum and minimizing soft tissue disruption. A bone cutter was used to create transverse osteotomy. No internal fixation was used. To minimize scarring, we used an intradermal buried suture technique with polydioxanone monofilament (PDS2; Ethicon, Somerville, NJ) for wound closure.

Then, the patients were placed in the prone position for Woodward procedure. A linear midline incision was made from the fourth cervical vertebra to the lower thoracic region, exposing the medial, superior, and inferior borders of the scapula. After carefully dissecting through the skin and subcutaneous tissues, the levator scapulae, trapezius, and rhomboid muscles were detached from their attachments on the spinous processes, while the omovertebral bar or any fibrous bands between the scapula and vertebra were excised. To facilitate repositioning, the latissimus dorsi and trapezius were detached 2–3 cm off the medial border of the scapula. The scapula was then mobilized, rotated in the coronal plane, and moved caudally until it was level with the contralateral scapula, verified by fluoroscopy. The scapula was finally repositioned with its inferior angle at the level of the 7th to 8th intercostal space and secured with nonabsorbable sutures into the surrounding muscles to ensure stability and anatomic alignment.

### 2.3 IONM methods

Continuous IONM was performed on all patients using a professional neurologic monitoring workstation (Cadwell Industries Inc., Cascade Systems, Kennewick, WA, USA). IONM included both MEPs and SEPs, monitored by a dedicated neurophysiologist. Subdermal electrodes for stimulation and recording were placed in the non-operative arm by the technician prior to disinfection. For the operative arm, electrode placement was performed by the surgeon after draping and surgical field preparation.

For MEP monitoring, transcranial electrical stimulation was applied via subdermal needle electrodes positioned over the motor cortex at 2 cm anterior to C3 and C4 based on the international 10–20 EEG system. Signals were recorded from key muscle groups deltoid, biceps, triceps, and abductor pollicis muscles. Stimulation parameters included a pulse count of 4–9, pulse width of 200∼500 *μ*s, inter-stimulus interval of 2 ms, and intensity of 250–400 V. Any decrease of 50% in MEP amplitude from baseline prompted an immediate response. MEPs were recorded at key surgical stages, including at the beginning of the prone position, after omovertebral bone resection, following muscle dissection, and after caudal displacement of the scapula. Total intravenous anesthesia was maintained throughout the operation with continuous monitoring of anesthesia depth. To prevent interference with IONM, the minimum alveolar concentration (MAC) of volatile anesthetics was kept below 0.5 (16) The contralateral arm was also monitored as a reference.

For SEP monitoring, stimulating electrodes were placed over the median and ulnar nerves, with recording electrodes placed at C3' and C4' following the international 10–20 system. Parameters included an intensity of 15–35 mA, a frequency of 2.7–5.1 Hz, pulse width of 200–500 μs, and a filter range of 30–3000 Hz, averaged over 200–500 stimulations. SEP signals were monitored every 10 min, with increased frequency during critical stages, such as scapula reduction. Alerts were defined by a 50% amplitude decrease or 10% latency prolongation. The contralateral arm was also monitored as a reference.

A dedicated neurophysiologist performed both SEP and MEP monitoring throughout the procedure. In the event of an alert during high-risk maneuvers (e.g., muscle dissection, omovertebral resection, and scapular caudal displacement), the neurophysiologist notified the surgeon, prompting an immediate pause in any mechanical manipulation. The operative extremity was returned to a neutral position, and protocol steps were activated to ensure patient safety, including verification of electrode positioning and adjustment of anesthesia. Amplitude levels were closely monitored for resolution before resuming the procedure.

### 2.5 Postoperative management

Postoperatively, patients were evaluated with plain radiographs immediately and at four weeks. Their arms were immobilized in a U-shaped cast for four weeks. Upon cast removal, shoulder abduction angles were recorded using a protractor, and active and passive range-of-motion (ROM) exercises began at home. Shoulder ROM was reassessed again at six weeks, and physiotherapy in the rehabilitation department was initiated if ROM did not improve sufficiently (less than 30 degrees of improvement after two weeks of home rehabilitation) to achieve maximum abduction and flexion. Follow-up examinations were conducted every three months during the first year and annually thereafter, during which cosmetic and functional outcomes were measured and documented. Patient satisfaction was assessed through a questionnaire survey, which was completed by the parents of the patients (Supplementary Appendix 1).

### 2.6 Statistical analysis

All statistical analyses were conducted using Stata version 16.0. Continuous variables are presented as mean ± standard deviation and analyzed using paired *t*-tests to determine statistical significance. Categorical variables were assessed using the Wilcoxon signed-rank test. A *P*-value of <0.05 was considered statistically significant.

## 3 Results

**Figure 1:**
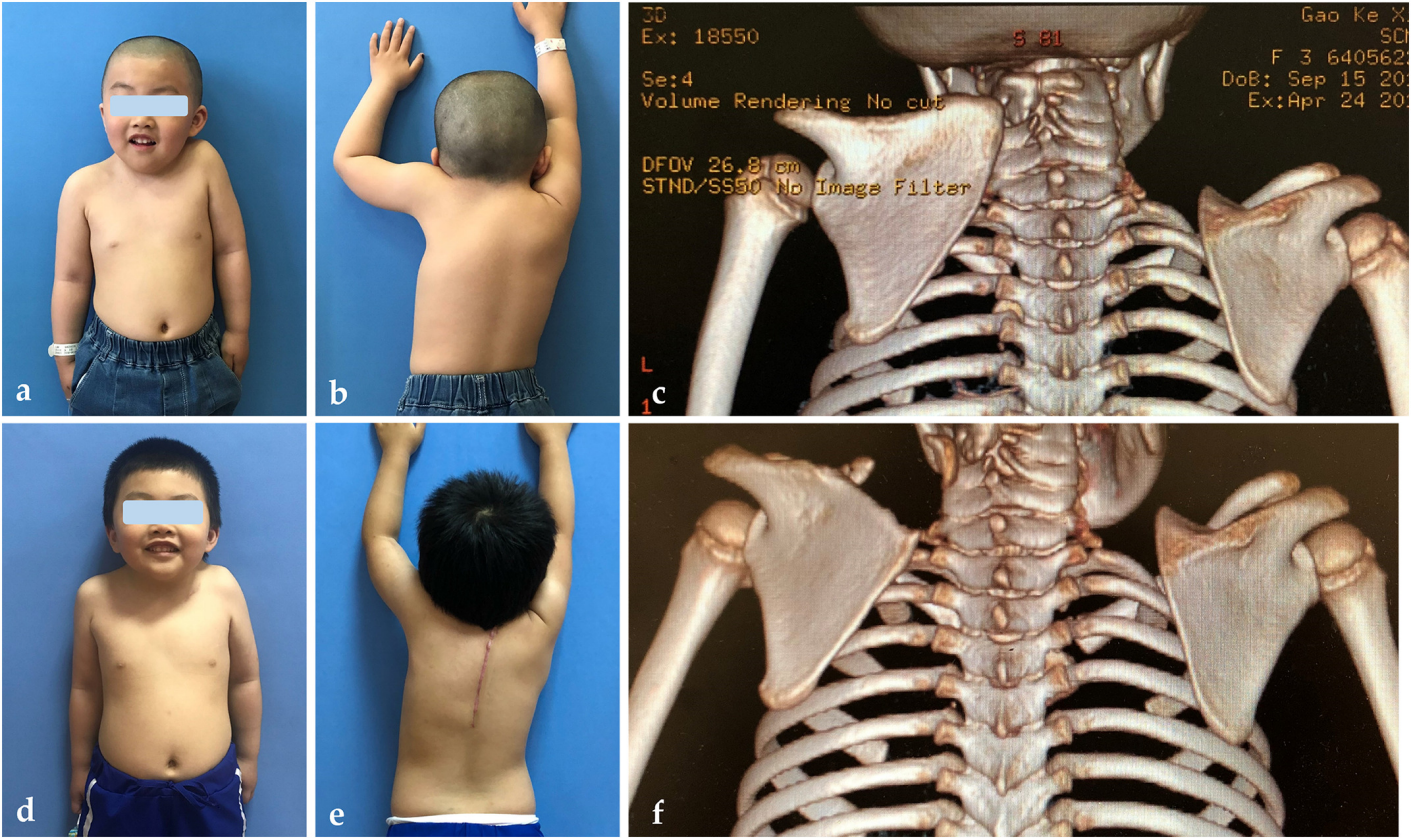
Preoperative presentation of a 4-year-and-1-month-old boy with left Sprengel deformity (Cavendish grade 4), showing appearance **(a)**, shoulder abduction **(b)**, and CT scan **(c)**. The patient underwent the Woodward procedure, leading to significant postoperative improvements in appearance **(d)**, shoulder abduction **(e)**, and radiographic outcomes **(f)**. Keloid formation was also observed.

The demographic characteristics of the patients are summarized in Table 1. A total of 36 patients (16 girls, 20 boys) underwent surgery at a mean age of 4.1 ± 1.4 years, with a mean follow-up of 5.6 years. Sprengel deformity was left-sided in 77.8% (*n* = 28) of the patients and right-sided in 22.2% (*n* = 8). Among these patients, 13 (36.1%) had an omovertebral bar. Preoperatively, 13 patients had Cavendish grade 3, and 23 had grade 4 deformities, with an average limited shoulder motion of 95.9° ± 15.5° (Table 1). Postoperatively, cosmetic appearance improved significantly from an average Cavendish grade of 3.4 ± 0.5–1.2 ± 0.4 (*P* < 0.05). The Rigault grade also improved markedly, with the average grade decreasing from 2.7 ± 0.5 preoperatively to 1.2 ± 0.4 postoperatively. Additionally, the mean shoulder abduction angle increased from 95.9° ± 15.5° to 169.6° ± 6.4° (*P* < 0.05), reflecting an approximately 74% improvement (Figure 1, Table 2).

**Table 1:** Characteristics of the study population (*n* = 36).

	Value
Age (yr)	4.1 ± 1.4 (2.1–8.3)
Gender	
Female	16 (44.4%)
Male	20 (55.6%)
Affected shoulder	
Right	8 (22.2%)
Left	28 (77.8%)
Omovertebral Bone	13 (36.1%)
Clavicle osteotomy	36 (100%)
Cavendish Grade	
III	23 (63.9%)
IV	13 (36.1%)
Rigault Grade	
II	10 (27.8%)
III	26 (72.2%)
Preop Abduction (angle)	95.9° ± 15.5°

Continuous data are given as the mean ± standard deviation with the range in parentheses. Categorical data are given as the count and percentage.

**Table 2:** Comparison of preoperative and postoperative abduction angles, Cavendish grades and rigault grades of the patients.

	Preoperative	Postoperative	Significance
Abduction angle	95.9 ± 15.5	169.6 ± 6.4	*P* < 0.001
Cavendish grade	3.4 ± 0.5	1.2 ± 0.4	z = 5.439
Rigault grades	2.7 ± 0.5	1.2 ± 0.4	z = 5.402

Abduction angle was analyzed with paired *t*-tests, Cavendish and Rigault grades were analyzed with Wilcoxon signed-rank test.

In terms of complications, we had one patient who experienced delayed wound healing with a superficial infection, which was successfully managed nonoperatively. Another patient developed a hidden hypertrophic scar, which resolved with conservative management. No cases of brachial plexus palsy, clavicular nonunion, vascular damage, clavicular pseudarthrosis, deep infection, scaphoid pterygoid, or deformity recurrence were reported. All clavicles on the surgical side completed reshaping and were in good condition three months post-operation. All parents were satisfied with the surgical outcomes.

During the scapular descent phase, two patients (5.6%) experienced a significant but temporary decrease in MEP amplitude on the operative side. In contrast, the contralateral IONM signals remained stable and unchanged in both cases. The patient's body temperature and blood pressure remained stable throughout the procedure. Supplementary Figure S1 provides detailed information for one of these patients, along with the corresponding IONM findings. Upon detecting these changes, the surgical team was promptly informed, allowing for immediate corrective measures. Specifically, the scapula was gradually released to alleviate tension on the brachial plexus. These interventions led to an obvious improvement in the signals, and no postoperative motor deficits were observed (Supplementary Figure S1**)**.

## 4 Discussion

This retrospective study analyzed the outcomes of 36 patients over a minimum follow-up period of five years, evaluating the impact of incorporating both intraoperative neuromonitoring (IONM) and clavicle osteotomy into the Woodward procedure. The results demonstrate significant improvements in both functional and cosmetic outcomes postoperatively, as shown by reduced Cavendish grades and a marked increase in shoulder abduction. Importantly, no neurovascular complications were observed. To our knowledge, this is the first study with a long-term follow-up period that utilized a combination of clavicle osteotomy and IONM for the Woodward procedure. Our findings provide valuable insights into the utility of IONM and clavicle osteotomy in enhancing the efficacy and safety of the surgery.

Intraoperative neuromonitoring (IONM) has been recognized as an essential component of spinal surgeries, particularly in high-risk procedures such as scoliosis correction, intradural tumor resections (17, 18), and selective dorsal rhizotomy for cerebral palsy (19). IONM enhances surgical safety by providing real-time feedback on nerve function, enabling immediate intraoperative adjustments in response to neurologic alerts and allowing for proactive measures to prevent nerve injury (20). Despite its growing recognition in shoulder deformity (SD) correction, the application of IONM in SD correction remains underexplored, with only a few studies involving limited patient numbers published to date. During the Woodward procedure for SD, IONM has proven to be a feasible and precise method for detecting intraoperative neurologic changes, potentially preventing brachial plexus injury. Feng et al. reported three cases of IONM alerts (10). One patient experienced an intraoperative decrease in motor evoked potential (MEP) signals, with only partial recovery following scapula readjustment, leading to incomplete paralysis of the operated arm. In contrast, the two patients who achieved full IONM signal recovery after scapula readjustment showed no signs of brachial plexus injury. Similar protective effects have been observed in previous studies (11, 21–23) (Table 3). These findings, consistent with our data, underscore the effectiveness of IONM in detecting neural incidents and preserving nerve integrity, thereby preventing brachial plexus injury. Notably, most IONM warnings were observed after scapular reduction, indicating that this surgical step poses a high risk for brachial plexus injury. Therefore, it should be executed with caution and in close collaboration with a neurophysiologist.

**Table 3:** Demographic characteristics and neurological outcomes of studies on correcting sprengel deformity using the woodward procedure and IONM with or without clavicle osteotomy.

Study no.	Author	Year	Study type	Patient no.	Clavicle osteotomy	IONM Method	Follow up	Neurological complications
1	Shea et al. (21)	2009	Case report	1	1 (100%)	SEP	24 months	A decrease in SEP signals was observed intraoperatively in the patient during scapula reduction, which resolved following repositioning of the scapula.
2	Ryabykh et al. (23)	2019	Retrospective study	18	5/18 (27.8%)	MEP	28 months	None
3	Pargas et al. (22)	2020	Case report	2	None	Not mentioned	2 months	A decrease in signal amplitude of the left deltoid muscle was observed intraoperatively during scapula reduction, which resolved after repositioning the scapula.
4	Majid et al. (25)	2021	Retrospective study	18	None	SEP MEP EMG EEG	12 months	None
5	Feng et al. (10)	2022	Retrospective study	46	11 (23.9%)	MEP SEP	12 months	A decrease in MEP signals occurred in 3 (6.5%) patients during scapular reduction. Following scapula repositioning, only one patient experienced a motor deficit due to brachial plexus injury, which resolved spontaneously within four months.
6	Li et al. (26)	2023	Case report	1	None	MEP SEP	3 months	None
7	Akar et al. (11)	2024	Retrospective study	18	18/18 (100%)	MEP SEP	18 months	A decrease in MEP signals was observed intraoperatively in only one case(5.6%) during scapula reduction, which resolved following repositioning of the scapula.

IONM, intraoperative neuromonitoring; SEP, somatosensory evoked potentials; MEP, motor evoked potentials; EMG, free-running electromyography; EEG, electroencephalogram.

The potential for enhanced surgical safety facilitated by intraoperative neuromonitoring (IONM) to complement postoperative improvements in shoulder function and cosmetic appearance has not been thoroughly studied. It is reasonable to speculate that the confidence provided by IONM allows surgeons to correct shoulder deformities more precisely within a safe range, potentially leading to greater improvements in both cosmetic and functional outcomes. Previous studies have reported shoulder function improvements ranging from 40 to 80° following the Woodward procedure (24). In our series of 36 shoulders, the mean increase was 74 degrees. Comparable results have been noted in other retrospective studies utilizing the Woodward procedure with IONM, showing shoulder abduction improvements of 68 degrees in 46 patients (10) and 67 degrees in 18 patients (11). Notably, a relatively lower increase of 48.3 degrees in 18 patients was also reported (25). Additionally, case reports documented a wide range of range of motion (ROM) improvements from 30° to 80° (21, 22, 26). The variation in improvement may be attributed to discrepancies in sample sizes, patient demographics, and confounding factors such as age, condition severity, and the surgeon's experience. Given these findings, there is not yet solid evidence to prove that IONM further improves shoulder range of motion or cosmetic results beyond what is achieved by the surgical procedure itself. Further research is needed to substantiate this hypothesis.

Although clavicle osteotomy has been recommended for Sprengel deformity (SD) correction for several decades (27), its necessity remains controversial. Theoretically, the clavicle is a component of the deformity, and its osteotomy supports the objectives of the Woodward procedure by not only providing additional space for scapular repositioning but also preventing compression of the brachial plexus during the inferior displacement of the scapula. Therefore, performing a clavicle osteotomy may allow better scapular repositioning and reduced risk of neurovascular injury. Until now, clavicle osteotomy is still being performed based on the surgeon's experience and preference. Some surgeons reserve clavicle osteotomies for patients over six years old, where more rigid periscapular tissues may limit scapular movement, or the patients who need extensive correction, such as those with Cavendish grades III or IV or severe Klippel-Feil syndrome (6, 7, 9, 28). Interestingly, brachial plexus injuries have been found to occur almost exclusively in cases where clavicle osteotomy was not performed (8, 29–31). This has raised concern and prompted surgeons to consider clavicle osteotomy more routinely. For instance, after experiencing two cases of complete brachial plexus palsy (Klippel-Feil syndrome, no clavicular osteotomy), Wada decided to routinely osteotomies clavicle in children over four years old, especially those with Klippel-Feil syndrome (9). After encountering a case (3 years and 3 months old, Cavendish grade III, no clavicle osteotomy) who developed radial pulsation loss and transient brachial plexus palsy, Naik suggested that clavicular osteotomy should be more liberally performed among Cavendish grade III and IV patients (8). With a case of complete plexus damage (2.5 years old, Rigault grade II, no clavicle osteotomy), Andrault et al. suggested that clavicular osteotomy should be performed systematically, regardless of the patient's age, when intraoperative monitoring is not available (31). In our study, all patients were Cavendish grade III and IV and underwent clavicle osteotomy, with no neurovascular complications observed in any of our cases. Similar results were reported by Gonen et al., who encountered no neurovascular impairment when performing clavicle osteotomy before applying the modified Green method, even in older patients (7). Therefore, clavicle osteotomy may help reduce the risk of neurovascular complications, especially in older patients with severe deformities. However, more studies are needed to further investigate this topic.

To date, no high-quality studies have compared the cosmetic and functional benefits of clavicle osteotomy between patients who undergo this procedure and those who do not. Comparing our results to Majid's study who underwent the Woodward procedure with only IONM (25), the addition of clavicle osteotomy seems to further improved postoperative cosmetic and functional results. Specifically, our average improvement in Cavendish scores (2.2 with a percentage change of 64.7%) is higher than Majid's results (1.8 with a percentage change of 58.3%). Similar findings were observed in the improvement of Rigault grade decrease (1.5 with a percentage change of 55.6% vs. 0.7 with a percentage change of 24.1%) and mean shoulder abduction angle (73.7° with a percentage change of 76.9% vs. 48.3° with a percentage change of 53.8%). We believe that our greater improvement in clinical results, with no neurologic complication incidence, could be attributed, at least partially, to the use of clavicle osteotomy during surgery. Our results align with that of Trisolino et al., who reported that adding a clavicular osteotomy to the modified Green procedure resulted in an average increase of 25° in postoperative shoulder abduction (2). Again, this conclusion should be interpreted with caution due to potential discrepancies such as sample sizes, patient demographics, and other confounding factors like patient age, severity of condition, and surgeon experience. We can only preliminarily conclude that clavicle osteotomy may help enhance postoperative cosmetic and functional improvement and reduce the risk of neurovascular complications. Therefore, we advocate for high-quality randomized controlled trials (RCTs) to further confirm these assumptions.

Based on our experience, we recommend clavicle osteotomy be routinely considered in SD correction. This relatively simple, quick procedure greatly alleviates postoperative anxiety for both surgeons and patients. Furthermore, thanks to the strong healing capacity and plasticity of young children's clavicles, we observed no complications such as nonunion, delayed healing, pseudarthrosis, or fracture. To promote optimal bone healing, we also emphasize the importance of careful periosteal protection during the osteotomy (32). Additionally, to minimize the risk of inadvertent neurovascular injury, we recommend using a bone cutter after drilling the anterior part of the clavicle with K-wires, rather than using an oscillating saw for clavicle osteotomy.

## 5 The limitations

We acknowledge several limitations in this study. Firstly, the single-center, retrospective nature of the study and the limited number of cases restrict our analysis and affect its statistical robustness. Larger, multicenter studies and high-quality randomized controlled trials (RCTs) are necessary to confirm the benefits of intraoperative neurophysiological monitoring (IONM), evaluate its cost-effectiveness, and establish standardized protocols for its use in Sprengel deformity surgery. Secondly, although clavicle osteotomy was consistently performed across our cases, further research is needed to confirm its independent contribution to clinical improvement and neurovascular protection in Sprengel deformity. Lastly, although we report an average follow-up duration of five years, extended follow-up beyond adolescence is essential to ascertain whether the functional and cosmetic improvements achieved in childhood persist into adulthood.

## 6 Conclusions

In conclusion, integrating the Woodward procedure with clavicle osteotomy and intraoperative neuromonitoring (IONM) offers a safe and effective method for correcting Sprengel deformity. We recommend that IONM and clavicle osteotomy be routinely considered in SD correction. Further research is necessary to independently validate their efficacy in this context.

## Data Availability

The raw data supporting the conclusions of this article will be made available by the authors, without undue reservation.

## Supplementary material

Supplementary Figure S1 Motor evoked potential (MEP) changes in a 5-year-old boy with left-sided Sprengel deformity during the Woodward procedure. A temporary decrease in signal amplitude of the left abductor pollicis was observed during scapular descent, with significant recovery following adjustment. No motor deficits were recorded postoperatively.

## References

[1] Farr S. Congenital and Acquired Deformities of the Pediatric Shoulder Girdle. Berlin: Springer (2022).

[2] Trisolino G Zarantonello P Todisco M Di Gennaro GL Menozzi GC Scheider P Patient-reported outcomes in children undergoing the modified green procedure for treating Sprengel’s deformity: results from a multicentric study. Children. (2024) 12(1):18. 10.3390/children12010018 39857850 PMC11763388

[3] Woodward JW. Congenital elevation of the scapula: correction by release and transplantation of muscle origins. J Bone Joint Surg Am. (1961) 43(2):219–28. 10.2106/00004623-196143020-00010

[4] Zarantonello P Di Gennaro GL Todisco M Cataldi P Stallone S Evangelista A Surgical treatment of sprengel’s deformity: a systematic review and meta-analysis. Children. (2021) 8(12):1142. 10.3390/children8121142 34943340 PMC8700527

[5] Chung SM Farahvar H. Surgery of the clavicle in Sprengle’s deformity. Clin Orthop Relat Res. (1976-2007). (1976) 116:138–41. 1277633

[6] Öner A Köksal A Çimen O Kargin D Albayrak A Akman YE. Modified woodward technique for Sprengel deformity and a modification of the cavendish classification. J Pediatr Orthop. (2020) 40(8):401–7. 10.1097/BPO.0000000000001582 32379247

[7] Gonen E Simsek U Solak S Bektaser B Ates Y Aydin E. Long-term results of modified green method in sprengel’s deformity. J Child Orthop. (2010) 4(4):309–14. 10.1007/s11832-010-0265-7 21804892 PMC2908338

[8] Naik P Chauhan H. Functional improvement in patients with Sprengel’s deformity following modified green’s procedure and simplified clavicle osteotomy—a study of forty cases. Int Orthop. (2020) 44(12):2653–63. 10.1007/s00264-020-04850-0 33094403

[9] Wada A Nakamura T Fujii T Takamura K Yanagida H Yamaguchi T Sprengel deformity: morphometric assessment and surgical treatment by the modified green procedure. J Pediatr Orthop. (2014) 34(1):55–62. 10.1097/BPO.0b013e318288b407 23774200

[10] Feng L Zhang X Guo D Li C Qi X Bai Y Utilization of intraoperative neuromonitoring during the woodward procedure for treatment of Sprengel deformity. J Shoulder Elbow Surg. (2022) 31(8):e405–e12. 10.1016/j.jse.2021.12.040 35121118

[11] Akar A Sever GB Akturk UD Serttas MF Ozdemir U Tekin OF Is the use of neuromonitoring necessary in Sprengel’s deformity surgery? J Pediatr Orthop B. (2024). 10.1097/BPB.0000000000001206 39229893

[12] Abuhassan FO. Subperiosteal resection of mid-clavicle in sprengel’s deformity correction. Strat Trauma Limb Reconstruc. (2011) 6:59–67. 10.1007/s11751-011-0115-2 PMC3150651 21773774

[13] Adkins G Pescador AM Koht A Gosavi S. Intraoperative neuromonitoring in intracranial surgery. BJA Educ. (2024) 24(5):173–82. 10.1016/j.bjae.2024.02.002 38646449 PMC11026914

[14] Jouve JL Choufani E Peltier E Khal A Pesenti S. Neuromonitoring for spine surgery in children. Orthop Traumatol Surg Res. (2024) 110(1):103780. 10.1016/j.otsr.2023.103780 38043606

[15] Wojtczak B Sutkowska-Stępień K Głód M Kaliszewski K Sutkowski K Barczyński M. Current knowledge on the use of neuromonitoring in thyroid surgery. Biomedicines. (2024) 12(3):675. 10.3390/biomedicines12030675 38540288 PMC10968482

[16] Kulkarni AM Shetty VL. Anesthesia considerations in patients undergoing spine surgery with evoked potential monitoring. J Spinal Surg. (2024) 11(2):56–63. 10.4103/joss.joss_10_24

[17] Fehlings MG Alvi MA Evaniew N Tetreault LA Martin AR McKenna SL A clinical practice guideline for prevention, diagnosis and management of intraoperative spinal cord injury: recommendations for use of intraoperative neuromonitoring and for the use of preoperative and intraoperative protocols for patients undergoing spine surgery. Global Spine J. (2024) 14(3_suppl):212s–22. 10.1177/21925682231202343 38526921 PMC10964898

[18] Daroszewski P Huber J Kaczmarek K Janusz P Główka P Tomaszewski M Real-time neuromonitoring increases the safety and non-invasiveness and shortens the duration of idiopathic scoliosis surgery. J Clin Med. (2024) 13(5):1497. 10.3390/jcm13051497 38592334 PMC10934752

[19] Xiao B Constatntini S Browd SR Zhan Q Jiang W Mei R. The role of intra-operative neuroelectrophysiological monitoring in single-level approach selective dorsal rhizotomy. Childs Nerv Syst. (2020) 36(9):1925–33. 10.1007/s00381-019-04408-5 31686140

[20] Skinner S Guo L. Intraoperative neuromonitoring during surgery for lumbar stenosis. Handb Clin Neurol. (2022) 186:205–27. 10.1016/B978-0-12-819826-1.00005-3 35772887

[21] Shea KG Apel PJ Showalter LD Bell WL. Somatosensory evoked potential monitoring of the brachial plexus during a Woodward procedure for correction of Sprengel’s deformity. Muscle Nerve. (2010) 41(2):262–4. 10.1002/mus.21545 20082410

[22] Pargas C Santana A Czoch WL Rogers KJ Mackenzie WG. Sprengel deformity in biological sisters. J Am Acad Orthop Surg Glob Res Rev. (2020) 4(4):e19.00120. 10.5435/JAAOSGlobal-D-19-00120 32377613 PMC7188265

[23] Ryabykh S Savin D Sayfutdinov M Ochirova P Gubin A Filatov EY Surgical treatment of Sprengel’s deformity using neurophysiological monitoring: analysis of a 7-year cohort. Genij Ortopedii. (2019) 25:550–4. 10.18019/1028-4427-2019-25-4-550-554

[24] Soldado F Barrera-Ochoa S Diaz-Gallardo P Nguyen TQ Nguyen DH Knörr J. Outcomes following endoscopic-assisted Woodward procedure for Sprengel deformity. J Child Orthop. (2021) 15(6):583–8. 10.1302/1863-2548.15.210138 34987669 PMC8670538

[25] Majid OB Alzaid SZ Al-Zayed Z Almonaie S Albekairi AA Ahmed M Outcomes of Woodward’s procedure for Sprengel’s shoulder using neurophysiological monitoring of the brachial plexus without clavicular osteotomy: a retrospective study. Cureus. (2021) 13(11):e19797. 10.7759/cureus.19797 34956786 PMC8692818

[26] Li H Zhang H Zhang X Yao Z Gao J Liu H Surgical treatment of severe Sprengel’s deformity: a case report. JBJS Case Connect. (2023) 13(1):e22.00648. 10.2106/JBJS.CC.22.00648 36928137

[27] Robinson R. The surgical importance of the clavicular component of Sprengel’s deformity. J Bone Joint Surg. (1967) 49:1481.

[28] Borges JL Shah A Torres BC Bowen JR. Modified Woodward procedure for Sprengel deformity of the shoulder: long-term results. J Pediatr Orthop. (1996) 16(4):508–13. 10.1097/01241398-199607000-00018 8784708

[29] Vuillermin C Wang KK Williams KA Hresko MT Waters PM. Sprengel’s deformity: an analysis of surgically and nonsurgically treated patients. J Shoulder Elbow Surg. (2021) 30(1):e1–9. 10.1016/j.jse.2020.04.018 33317707

[30] Grogan D Stanley E Bobechko W. The congenital undescended scapula. Surgical correction by the Woodward procedure. J Bone Joint Surg Br. (1983) 65(5):598–605. 10.1302/0301-620X.65B5.6643564 6643564

[31] Andrault G Salmeron F Laville JM. Green’s surgical procedure in Sprengel’s deformity: cosmetic and functional results. Orthop Traumatol Surg Res. (2009) 95(5):330–5. 10.1016/j.otsr.2009.04.015 19648073

[32] Ropars M Thomazeau H Huten D. Clavicle fractures. Orthop Traumatol Surg Res. (2017) 103(1s):S53–s9. 10.1016/j.otsr.2016.11.007 28043849

