# Supplementary figures and images for: Woodward procedure with intraoperative neuromonitoring for Sprengel deformity: a retrospective study with a mean 5-year follow-up
Source: Front Pediatr. 2025 Jun 27;13:1541132. doi: 10.3389/fped.2025.1541132 (PMC12245842; doi:10.3389/fped.2025.1541132)

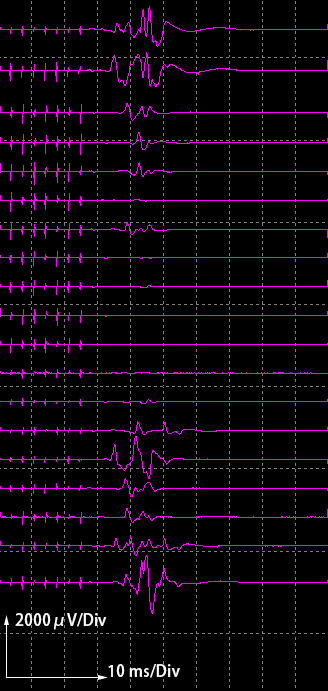

Supplement: Supplementary Figure S1 — Motor evoked potential (MEP) changes in a 5-year-old boy with left-sided Sprengel deformity during the Woodward procedure. A temporary decrease in signal amplitude of the left abductor pollicis was observed during scapular descent, with significant recovery following adjustment. No motor deficits were recorded postoperatively. [file Image1.tif]
