# Supplementary material for: Woodward procedure with intraoperative neuromonitoring for Sprengel deformity: a retrospective study with a mean 5-year follow-up
Source: Front Pediatr. 2025 Jun 27;13:1541132. doi: 10.3389/fped.2025.1541132 (PMC12245842; doi:10.3389/fped.2025.1541132)
Supplement: Supplementary file 2 [file Datasheet1.pdf]

Questionnaire

Instructions: Please answer the following questions based on your observations and experiences regarding your child's condition after undergoing the Woodward procedure for Sprengel deformity. Your responses will help us improve patient care and outcomes. All responses will be kept confidential and will be used solely for scientific research purposes.

Section A: Participant Information

1. Child's Name: ___________________
2. Child's Hospital ID: ______________
3. Parent's Name: __________________
4. Parent's Relationship to Child: [ ] Mother [ ] Father [ ] Other (please specify): _________
5. Time Since Surgery: ______ months/years

Section B: Cosmetic Outcomes

1. How satisfied are you with the overall appearance of your child's shoulder and back after surgery?
    - Very Satisfied
    - Satisfied
    - Neutral
    - Dissatisfied
    - Very Dissatisfied
2. How would you rate the symmetry between your child's shoulders after the procedure?
    - Excellent
    - Good
    - Fair
    - Poor
    - Very Poor
3. How noticeable is the surgical scar?
    - Not noticeable
    - Slightly noticeable
    - Moderately noticeable
    - Very noticeable
    - Extremely noticeable

Section C: Functional Outcomes

4. How satisfied are you with your child's range of motion in the affected shoulder?
    - Very Satisfied
    - Satisfied
    - Neutral
    - Dissatisfied
    - Very Dissatisfied
5. How would you rate your child's ability to perform daily activities post-surgery?
    - Excellent
    - Good
    - Fair
    - Poor
    - Very Poor

6. Have you noticed any improvement in your child's physical activity levels since the surgery?
    - Significant improvement
    - Moderate improvement
    - Slight improvement
    - No improvement
    - Deterioration

Section D: Overall Satisfaction

7. Overall, how satisfied are you with the outcomes of the surgery?
    - Very Satisfied
    - Satisfied
    - Neutral
    - Dissatisfied
    - Very Dissatisfied
8. Would you recommend our department to other parents with children suffering from Sprengel deformity?
    - Definitely
    - Probably
    - Not sure
    - Probably not
    - Definitely not

Section E: Additional Comments

9. Please provide any additional comments or suggestions regarding your child's treatment and outcomes:

____

____

____

End of Questionnaire

Thank you for taking the time to complete this questionnaire. Your feedback is invaluable to us.
